# Fluoroquinolones directly drive mitochondrial hyperpolarization and modulate iNOS expression in monocyte-derived macrophage populations

**DOI:** 10.1093/discim/kyaf018

**Published:** 2025-11-12

**Authors:** Alexander W Hardgrave, Megan Dooley, Ivy Maminimini, Adura Faniyi, Antonia Christodoulidou, Yasmine Alshammari, Helen J March, Riccardo V D’Elia, John J Worthington

**Affiliations:** Faculty of Health and Medicine, Department of Biomedical and Life Sciences, University of Lancaster, Lancaster, UK; Faculty of Health and Medicine, Department of Biomedical and Life Sciences, University of Lancaster, Lancaster, UK; Faculty of Health and Medicine, Department of Biomedical and Life Sciences, University of Lancaster, Lancaster, UK; Faculty of Health and Medicine, Department of Biomedical and Life Sciences, University of Lancaster, Lancaster, UK; Faculty of Health and Medicine, Department of Biomedical and Life Sciences, University of Lancaster, Lancaster, UK; Faculty of Health and Medicine, Department of Biomedical and Life Sciences, University of Lancaster, Lancaster, UK; Faculty of Allied Health Sciences, Department of Medical Laboratory Sciences, Kuwait University, Kuwait City, Kuwait; Pharmacy Department, Royal Oldham Hospital, Northern Care Alliance NHS Trust, Oldham, UK; CBR Division, Defence Science and Technology Laboratory Porton Down, Salisbury, UK; Strathclyde Institute of Pharmacy & Biomedical Sciences, University of Strathclyde, Glasgow, UK; Faculty of Health and Medicine, Department of Biomedical and Life Sciences, University of Lancaster, Lancaster, UK

**Keywords:** macrophage, lung, mucosal immunology, antibiotics, mitochondria

## Abstract

**Introduction:**

The fluoroquinolone levofloxacin is often selected for use prophylactically as well as during respiratory infections. However, studies on how these antibiotics may alter innate immunity, as opposed to their bactericidal activity, are limited.

**Materials & Methods:**

We employed a murine model of therapeutically relevant antibiotic dosing to investigate the effect of prophylactic levofloxacin treatment on innate immunity.

**Results:**

We observed mild pathology at the barrier sites of both the lung and colon in terms of alveolar space and goblet cell numbers, respectively. Although we saw no alteration in lung immune populations of neutrophils, eosinophils, or dendritic cells, we did see heightened expression of macrophage inducible nitric oxide synthase (iNOS). Interestingly this was only present in the shorter-lived CD206− interstitial macrophage subset and not observed in the long-lived resident alveolar population. Within the large intestine levofloxacin also targeted iNOS expression in the shorter-lived TIM4-CD4+ population but conversely inhibiting expression in the microbially rich colon. We therefore utilized the bone marrow-derived macrophage system, devoid of microbial interactions and demonstrated that levofloxacin had a direct effect on driving iNOS expression and increasing phagocytosis but only when present in developing macrophages and not mature macrophage populations. Our macrophage observations were replicated in ciprofloxacin, but not doxycycline-treated animals, indicating a fluoroquinolone specific action. Mechanistically, fluoroquinolone treatment was associated with mitochondrial hyperpolarization, indicating a direct alteration of macrophage immunity via off target effects.

**Conclusion:**

Collectively, this study demonstrates a direct action of fluoroquinolones on macrophage immunity, which should be considered when selecting antibiotics for tissue specific and prophylactic use.

## Graphical Abstract

**Graphical Abstract:**
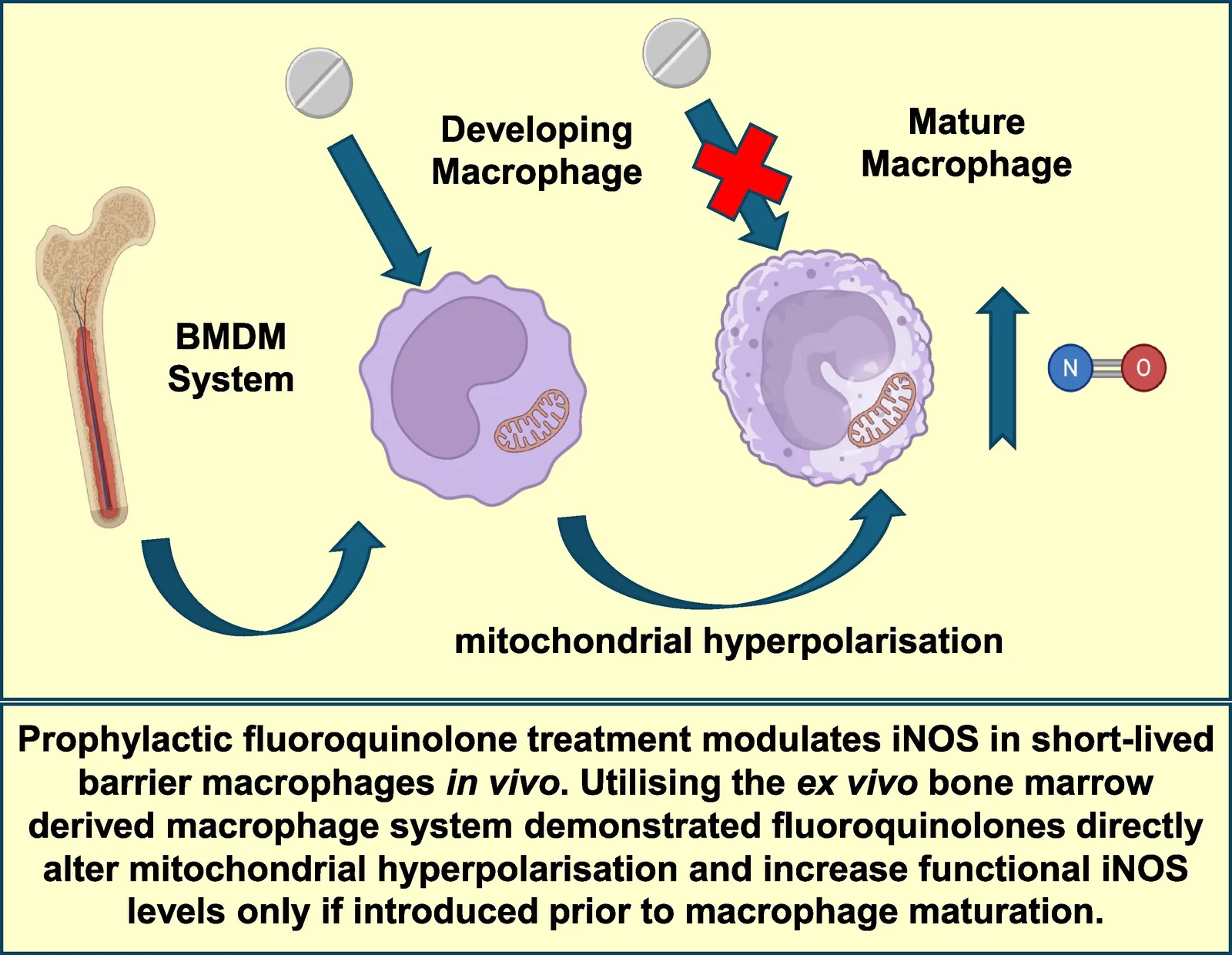

## Introduction

The lung must balance the efficient exchange of oxygen and carbon dioxide with the continuous risk of airborne pathogens. The lung therefore hosts a myriad of immune cells designed to protect us from these invaders, but these defences can still be overwhelmed, resulting in life-threatening infections. Indeed, lower respiratory tract infections were ranked as the fifth leading cause of death both globally and within the UK [1]. Antibiotics are the main treatment for limiting serious complications in many bacterial infections [2] and act by inhibiting the growth or killing infective bacteria [3]. However, antibiotics do not selectively target pathogenic bacteria and also affect our microbiota [4, 5] as well as directly influencing host cells, which collectively may drive side effects [6]. The immune system is not impervious to the off-target actions of antibiotics, and this can result in both beneficial and adverse effects [7–9].

Macrophages are key cells in the innate immune response to respiratory infections [10], performing many functions, ranging from phagocytosing pathogens and clearing cell debris to antigen presentation and cytokine release. Whole-genome transcriptional profiling of the lung following antibiotic treatment highlights alterations in macrophage-related signalling pathways [11], indicating this immune population as particularly susceptible to antibiotic influence. Indeed, recent findings have uncovered several mechanisms of antibiotic-induced macrophage dysfunction *in vivo*. This includes the downturn in microbially derived short-chain fatty acid metabolites, whose macrophage-tolerizing ability is impacted during antibiotic-induced intestinal microbiota dysbiosis [12]. Antibiotic-induced intestinal dysbiosis can also impact macrophages at distal sites, with reduced phagocytosis of *Burkholderia pseudomallei* observed during melioidosis [11]. Metabolic alterations are also not limited to the microbiome, as antibiotic treatment of germ-free mice impacts host cell metabolites such as adenosine monophosphate, which in turn inhibits local macrophage phagocytic killing [13]. Finally, antibiotics can directly influence macrophage function via inducing metabolic reprogramming in both a mitochondrial dependent [13] and independent fashion [14].

In this study, we chose to investigate the potential for levofloxacin, a third-generation fluoroquinolone, to modulate the innate immune system utilizing a 2-week dosing regime in mice. Our results show that fluoroquinolones induce mild tissue pathology in both the lung and intestine as well as modulating expression of macrophage inducible nitric oxide synthase (iNOS). Interestingly, this occurred in shorter-lived macrophage populations, mirroring our *in vitro* bone marrow-derived macrophage (BMDM) studies demonstrating that levofloxacin drove iNOS expression and increased phagocytosis in developing but not matured macrophages. Although we did not see any alterations in monocyte populations in either tissue, we did detect mitochondrial hyperpolarization in our fluoroquinolone-treated BMDMs, a crucial factor in inflammatory macrophage polarization [15]. Collectively, these data suggest fluoroquinolones may benefit pathogen clearance via increasing macrophage iNOS levels, but this may be at the expense of local tissue pathology. Moreover, our findings provide new insights into the direct modulation of innate immunity by fluoroquinolones and may inform potential therapeutic approaches, including site of infection and prophylactic use.

## Materials and methods

### Ethics statement

All mice were group housed in individually ventilated cages on a 12-hour light-dark cycle (lights on at 07:00) at 22 ± 1°C and 65% humidity. All procedures were carried out in accordance with the U.K Animals (Scientific Procedures) Act (1986). Experiments were carried out under UK Home Office project licences 70/8521 and PP4157153. All experiments conformed to the relevant University Animal Welfare and Ethics Review Board and Animal Research: Reporting of *in vivo* Experiments guidelines. All personnel were specifically trained for handling mice in the Physiological Service Unit, and all procedures were completed by trained and experienced researchers.

All animals were observed daily, and their weight was monitored at weekly (daily during pilot experiments) intervals. If an animal lost ≥20% of body weight compared to untreated controls, schedule one would be performed. Schedule one would also be performed at any point during the procedure if there was any indication of ill health, such as hunching, ‘gurning’, piloerection or rectal bleeding.

### Animals

C57BL/6 wild-type animals were purchased from Envigo, UK and housed under specific pathogen-free conditions at Lancaster University. Experiments were performed using age- and sex-matched mice at 11–14 weeks of age. Mice were provided sterilized water and chow *ad libitum* and acclimatized for a minimum of 1 week prior to experimental manipulation. Experimenters were blind to the treatment conditions during analysis of the animals, with this information revealed at the point of statistical data analysis.

### *In vivo* antibiotic treatments

Mice received ciprofloxacin, levofloxacin or doxycycline at doses of 100 mg/kg. Animals were dosed at approximately 10am and 6pm every day via 100 µl oral gavage. Non-antibiotic control mice received a vehicle mock dose of qH_2_0 for fluroquinolones or phosphate-buffered saline (PBS) for doxycycline.

### Histology

Mouse lung tissues were inflated and left lobe removed, while 1 cm of colon was removed before samples were fixed in Carnoys buffer (60% absolute ethanol, 30% Chloroform, 10% glacial acetic acid) for 4 hours and embedded in paraffin wax via dehydrating down an alcohol gradient (1 hour × 90% ethanol, 2 × 1 hour 100% ethanol), followed by 2 × 45 minutes in Xylene and 40 minutes/overnight in molten paraffin wax (Fisher). 5 μm tissue sections were placed on slides and dewaxed in xylene for 10 minutes before being passed through an alcohol gradient (100%, 90%, 70%, 50%, H_2_O). Slides were then stained for goblet cells in alcian blue (Sigma) for 5 minutes, 1% periodic acid (Thermo Scientific) for 5 minutes, then Schiff’s reagent (Thermo Scientific) for 15 minutes. Slides were then counterstained with haematoxylin (Thermo Scientific) for 1 minute. Slides were thoroughly washed between each stain. Once stained, slides were dehydrated via an ethanol-xylene gradient (70% ethanol for 30 seconds, 100% ethanol for 30 seconds, then xylene for 1 minute) and coverslipped using DPX (Sigma). Images were captured using Nikon Eclipse E600 LED microscope and analysed using ImageJ Fiji software. Goblet cells were quantified in the colon via counting 20 random colon crypt units and in lung sections were counted at 400× magnification of images of the large airways and averaged across five random images of the large airways per lung. For structural analysis of lung sections, images were converted into black and white using the threshold function and inverted. The analyse particle function was then used to quantify space.

### Tissue digestion

The right lobe of the lung was finely diced using scissors and placed in 2 ml lung digest medium (PBS (Sigma), 0.1 mg/ml Liberase TM (Roche), 50 µg/ml DNAse I (Roche)) for 30 minutes in a vigorously shaking incubator at 37°C. Digestion was stopped after 30 minutes by adding 5 mM ethylenediaminetetraacetic acid (EDTA) (Fisher). Digestion medium was passed through a 100 µm cell strainer to produce a single-cell suspension. If required, red blood cell lysis was then performed by resuspending in RBC lysis buffer (Sigma) for 1 minute at room temperature (RT). An equal volume of RPMI was added to stop lysis. Colons were excised and lamina propria lymphocytes were prepared as described [16]. Cells were then centrifuged at 400 g for 5 minutes at 4°C and resuspended in 1 ml RPMI full media [10%FCS (Gibco), 1% Pen/Strep (Sigma), 1% L-glutamine (Sigma), 1% non-essential amino acids (Sigma), 1% Hepes (Sigma), and 50 µM β-mercaptoethanol (Sigma)].

### Flow cytometry

Cell counts were carried out using a Countess Automated Cell Counter (Invitrogen) and disposable counting slides (NanoEnTek) to determine the cellularity of each sample. Samples were diluted 1/10 in media if required, and diluted 1/2 in 0.4% Trypan blue (NanoEnTek). Cells were then stained with antibodies using the eBioscience Foxp3 permeabilization kit (Cat No.00-5523-00) according to the manufacturer’s instructions, with 30 minutes for fixation at 4°C.

Cell suspensions were blocked with anti-FcγR Ab (clone 24G2; eBioscience, Cat No. 14-0161-82) at 1:200 dilution for 20 minutes at 4°C before labelling with combinations of antibodies specific for Ly6G (clone 1A8; Biolegend, Cat No. 127606), Siglec F (clone E50-2440; BD, Cat No. 562757), CD11c (clone N418; Biolegend, Cat No. 117324), CD45 (clone 30-F11; Biolegend, Cat No. 103134), Ly6C (clone HK1.4; Invitrogen, Cat No. 45-5932-82), CD11b (clone M1/70; Biolegend, Cat No. 101212), IA/IE (clone M5/114.15.2, Biolegend, Cat No. 107622), CD64 (clone 027; Invitrogen, Cat No. MA5-46783), CD4 (clone GK1.5; Bioegend, Cat No. 100414), iNOS (clone CXNFT; eBioscience, Cat No. 53-5920-82), CD206 (clone C068C2; Biolegend, Cat No. 141727), F4/80 (clone BM8; Biolegend, Cat No. 123146), and Tim-4 (clone RMT4-54; BioLegend, Cat No. 130006). Antibody panels were used at 1.5 μg/ml for surface markers and 3 μg/ml for intracellular markers and incubated for 30 minutes at 4°C. Fluorescence minus one stains were utilized for iNOS staining.

All samples were analysed on a Beckman Cytoflex or Sony MA900 and analysed with FlowJo Software (TreeStar) and numbers calculated utilizing cellularity counts. Cells were gated for singlets and immune cells were gated on CD45, with myeloid cells identified utilizing gating as previously described for lung [17] and gut [18] with the addition of macrophage subset markers CD206, CD4, TIM4, and iNOS as indicated.

### Bone marrow isolation and macrophage derivation culture

Femurs and tibias of mice were removed and cleaned in a sterile changing station. The bone marrow was flushed out of the bone using a 25 g needle and syringe containing 2.5 ml of Dulbecco's modified Eagle medium (DMEM) w/10% Foetal Calf Serum (FCS) (Gibco and Sigma) and homogenized by passing through a 100 µm cell strainer (Fisher). Cell suspension was pooled and resuspended in red blood cell lysis buffer at RT (Sigma). After 2 minutes, DMEM w/10% FCS was added, and cells were centrifuged at 400 g for 5 minutes at 4°C and resuspended in 1 ml DMEM w/10% FCS.

For culture, cells were plated out in 6 well plates (Corning) at 1 × 10^6^ cells per well in 4 ml full BMDM medium (DMEM w/GLUTAMAX, 10% FCS, 20 ng/ml Macrophage Colony Stimulating Factor (M-CSF) and cultured at 37°C in 5% CO_2_. On Days 4 and 6 of incubation, 2 ml of medium was removed from each well, and replaced with fresh medium. On Day 7 of incubation, cells were ready for analysis or further treatment.

### Bone marrow-derived macrophage polarization

Media and cells were removed from each well, and 2 ml pre-warmed PBS w/3 mM EDTA and 10 mM Glucose added to aid detaching adherent cells. Plates were returned to the incubator for 15 minutes. Cells were agitated and the cell suspension removed with a micropipette. Cells were replated out in BMDM media and allowed to settle and then stimulated with 5, 20, or 40 ng/ml Interferon Gamma (IFNy) (Peprotech) as indicated.

### IgG phagocytosis assay

IgG FITC-labelled beads (Cayman Chemical) were added to BMDM suspensions to a final dilution of 1/500. Cells were then incubated for 60 minutes at 37°C in 5% CO_2_ or at 4°C. To remove background FITC fluorescence from surface-bound beads, cells were quenched in trypan blue quenching solution (Cayman Chemical) for 2 minutes. Cells were then centrifuged (400 g for 5 minutes at 4°C) and washed in PBS (1% (Bovine serum albumin) BSA), before analysis via flow cytometry.

### Lactate dehydrogenase cytotoxicity assay

Before performing the assay, it was optimized according to the manufacturer’s instructions to determine the appropriate concentration of cells per well. 100 µl BMDMs were plated in BMDM culture media into a 96-well plate at 1 × 10^4^ cells/well and incubated overnight at 37°C in 5% CO_2_. Cells were incubated for 45 minutes with 10 µl of fluoroquinolone to a desired range of final concentrations. Additional cells were incubated with 10 µl qH_2_O (spontaneous lysis control) or 10 µl 10× lysis buffer (maximum lysis control) (Thermo).

After incubation, 50 µl of sample medium was transferred from each well into a separate corresponding plate. 50 µl of reaction Mixture (Thermo) was then added to each well and mixed. The plate was then incubated in the dark for 30 minutes at RT, after which 50 µl of stop solution (Thermo) was added to each well.

Absorbance was then measured at 490 nm (680 nm reference) using an Infinite M200 pro plate reader (Tecan). To calculate LDH activity, 680 nm absorbance was subtracted from 490 nm absorbance.

### Greiss assay

Nitric oxide production was determined by measuring its stable end product nitrite, using a Griess reagent (Thermo) according to manufacturer’s protocol. Briefly, 130 μl of supernatant was added to 96-well plate, followed by 30 μl Griess Reagent and 150 μl dH_2_O for 30 minutes at RT. Absorbance at 540 nm was measured by microplate reader and nitrite concentrations were estimated using a standard nitrite curve.

### Mitochondrial staining

Samples were centrifuged at 400 g for 5 minutes at RT and resuspended in MitoTracker Green FM and MitoTracker Orange CMTMRos, working conc: 250 and 100 nM respectively (Invitrogen) and incubated at 37°C for 30 minutes. The samples were then centrifuged at 400×g at RT and resuspended in 200 µl PBS (1% BSA) and transferred into FACS tubes. Samples were then analysed on a Cytoflex flow cytometer (Beckman Coulter).

### Statistical analysis

Results are expressed as mean ± SEM. Where statistics are quoted, normality was assessed with the Shapiro–Wilk test and two experimental groups were compared via Student’s *t* or Mann–Whitney test for parametric and non-parametric data, respectively as indicated. Three or more groups were compared with ANOVA with Tukey’s or Dunnetts *post-hoc* test or Kruskal–Wallace with Dunn’s *post-hoc* test for parametric and non-parametric data, respectively as indicated. A *P* value of <0.05 was considered statistically significant. *P < 0.05, **P < 0.01, and ***P < 0.005 for indicated comparisons.

## Results

### Levofloxacin treatment results in lung pathology and increased iNOS expression in the interstitial CD206− macrophage population

**Figure 1.**
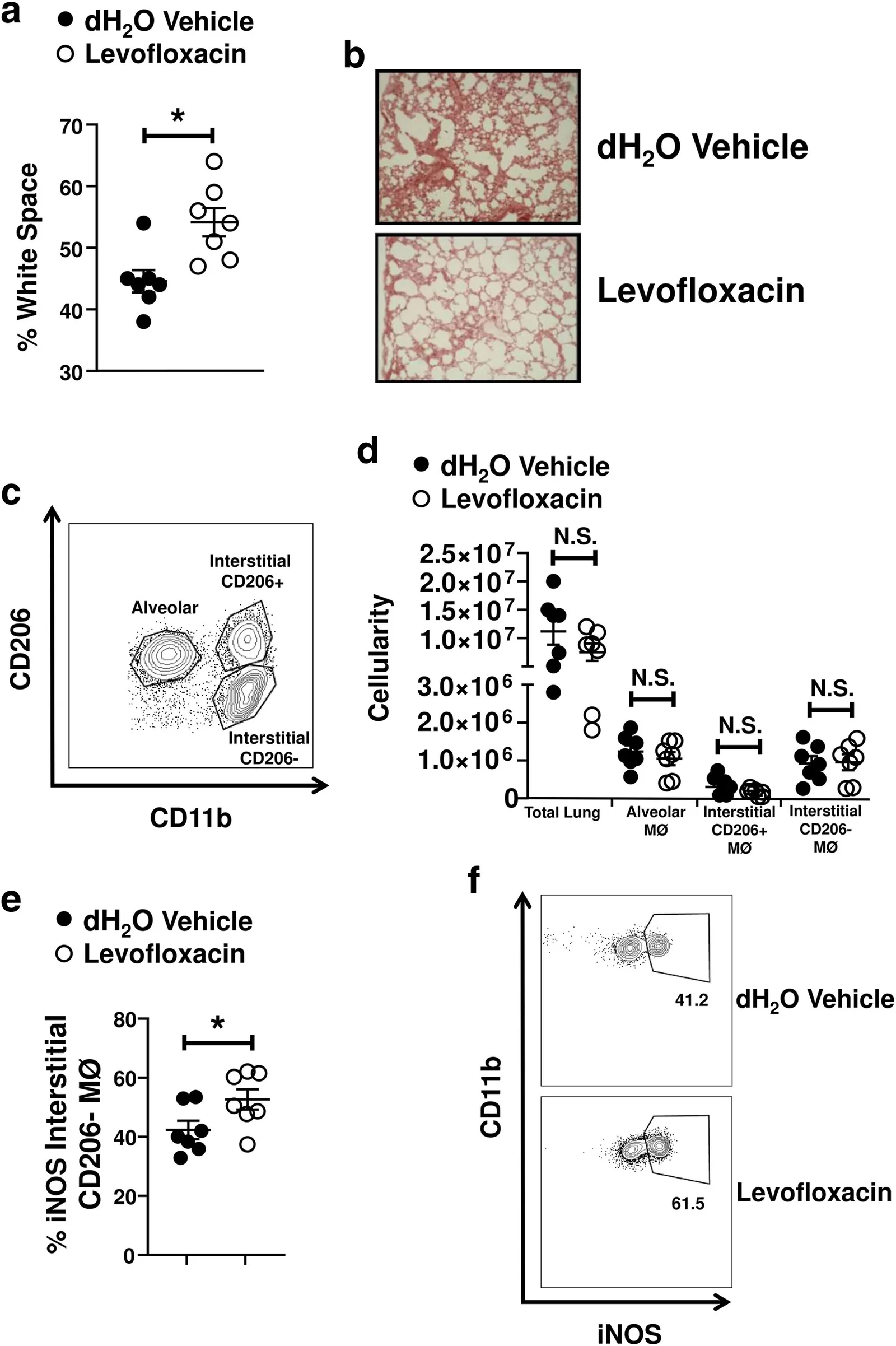
Levofloxacin drives iNOS expression in the lung interstitial CD206− macrophage population. C57BL/6 mice were gavaged twice daily with either 100 mg/kg levofloxacin or dH_2_O mock dose for 14 days. White space of lungs (a) and representative H&E-stained histology images of lung (b) following harvest at 14 days of treatment. (c) Identification of alveolar and interstitial macrophage lung populations via flow cytometry and (d) total cellularity of lung and macrophage populations. Proportion of lung interstitial CD206− macrophages expressing iNOS (e) and representative flow cytometry plots (f). Data (*n* = 7 mice per group) are mean ± SEM from three independent experiments performed. *P < 0.05; **P < 0.01; ***P < 0.005; N.S., not significant via ANOVA followed by Dunnett’s multiple comparison test (a and e) or Kruskal–Wallace followed by Dunn’s multiple comparison test (d) for indicated comparisons between groups.

C57BL/6 mice were dosed twice daily with 100 mg/kg of the fluoroquinolone levofloxacin or H_2_O control dose via oral gavage for 14 days to mimic a prophylactic therapeutically relevant high dose [19–21] and then lung tissue was examined. Histological staining of lung tissue demonstrated significantly increased lung alveolar space pathology following levofloxacin treatment as compared to H_2_O control dose (Fig. 1a and b), although no alteration was seen in goblet cell number in bronchioles between the treatments (Supplementary Figure S1a). We next analysed key myeloid innate cell populations in the lung tissue via flow cytometry to assess if any changes occurred following levofloxacin treatment. There was no alteration in overall cellularity in the lung in levofloxacin-treated animals as compared to H_2_O-dosed controls (Fig. 1d). Moreover, we did not see any alteration in neutrophilia, eosinophilia or changes in dendritic cell numbers in levofloxacin-treated animals as compared to H_2_O-dosed controls (Supplementary Figure S1b). Given the importance of macrophages in lung bacterial infections, we distinguished between the alveolar macrophage and interstitial macrophage populations [17, 22], further subdividing interstitial macrophages into perivascular and nerve-associated niche populations via CD206 expression [23 ] (Fig. 1c) but did not see any differences in alveolar or interstitial macrophage subset numbers following levofloxacin treatment (Fig. 1d).

Examining the expression of the pro-inflammatory inducible nitric oxide synthase (iNOS) within the tissue-resident alveolar macrophage population, we saw no alteration in levofloxacin-treated groups as compared to H_2_O-dosed controls (Supplementary Figure S1c). However, in the CD206− interstitial macrophage population, we saw a significant increase in iNOS-positive macrophages in levofloxacin-treated animals as compared to controls (Fig. 1e and f). This appeared to be specific to the CD206− population, as CD206+ population remained unchanged between treatments (Supplementary Figure S1d).

Collectively, these data indicate that prophylactic levofloxacin treatment results in mild lung pathology and importantly an increase in iNOS expression in the short-lived interstitial CD206-macrophage population.

### The fluoroquinolone ciprofloxacin recapitulates the lung macrophage iNOS expression of levofloxacin

**Figure 2.**
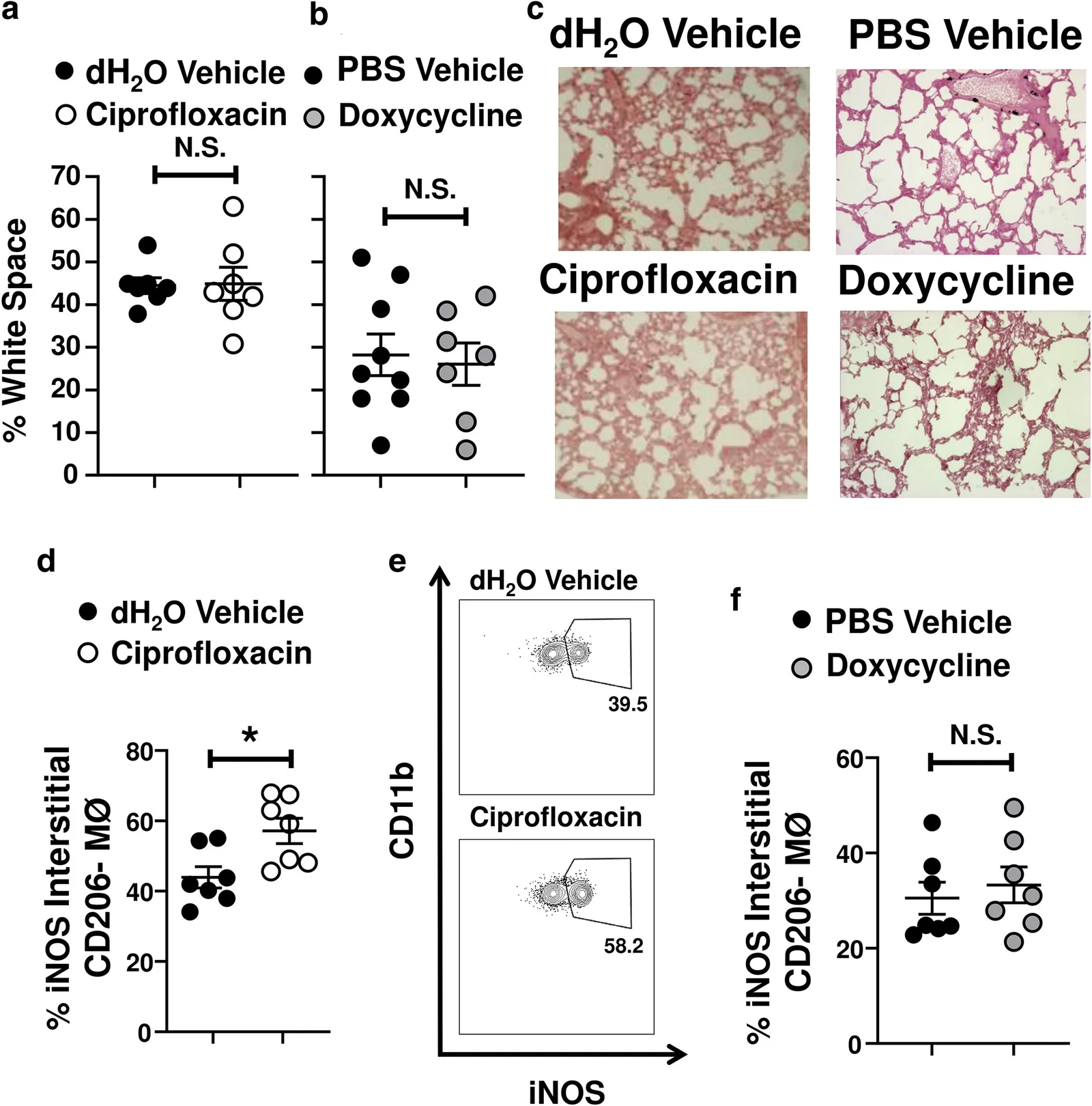
Ciprofloxacin, but not doxycycline, drives iNOS expression in the lung interstitial CD206− macrophage population. C57BL/6 mice were gavaged twice daily with either 100 mg/kg levofloxacin or doxycycline or dH_2_O/PBS mock dose for 14 days. White space of lungs (a and b) and representative H&E-stained histology images of lung (c) following harvest at 14 days of treatment. Proportion of lung interstitial CD206− macrophages expressing iNOS following ciprofloxacin treatment (d) and representative flow cytometry plots (e). Proportion of lung interstitial CD206− macrophages expressing iNOS following doxycycline treatment (f). Data (*n* = 7 mice per group) are mean ± SEM from three independent experiments performed. *P < 0.05; **P < 0.01; ***P < 0.005; N.S., not significant via ANOVA followed by Dunnetts’s multiple comparison test (a, d) or Student’s *t*-test (b and f), for indicated comparisons between groups.

We concurrently tested whether this was unique to the fluoroquinolone class of antibiotics, also examining lung macrophage populations in mice treated with ciprofloxacin, a second-generation fluoroquinolone, and completed additional experiments using doxycycline, a tetracycline class of antibiotic. We again examined lung histology, but in contrast to levofloxacin (Fig. 1a and b), ciprofloxacin-treated mice demonstrated no alteration in alveolar space as compared to vehicle controls (Fig. 2a and c). Similarly, doxycycline-treated mice demonstrated no lung pathology at the histological level as compared to vehicle controls (Fig. 2b and c). Examining the total cellularity of the lung and macrophage populations demonstrated that ciprofloxacin did not alter the total cellularity, nor number of alveolar, interstitial CD206+ or CD206− macrophage subsets (Supplementary Figure S2a). Consistent with its fluoroquinolone counterpart levofloxacin, ciprofloxacin treatment, although not influencing iNOS expression in the alveolar and CD206+ interstitial macrophage population (Supplementary Figure S2b and c), drove a significant increase in iNOS expression in the CD206− interstitial lung macrophage population as compared to vehicle controls (Fig. 2d and e). Finally, we examined the potential for fluoroquinolones to alter other lung immune cell iNOS expression in neutrophil, eosinophil, DC, and B-cell populations. However, ciprofloxacin treatment did not significantly alter iNOS expression in any of these immune populations as compared to vehicle controls (Supplemnetary Figure S2d).

In contrast to these fluoroquinolone treatments, doxycycline did not significantly change iNOS expression in the CD206− interstitial macrophage population, with levels comparable to vehicle controls (Fig. 2f). Indeed, overall cellularity, macrophage subset number and alveolar and CD206+ macrophage iNOS expression were also unaltered by doxycycline treatment as compared to vehicle controls (Supplementary Figure S2e, f and g).

Collectively, these data indicate antibiotic-induced increase in iNOS expression in the short-lived interstitial CD206− macrophage population appears unique to the fluoroquinolone group of antibiotics.

### Fluroquinolones, but not doxycycline treatment, results in altered iNOS expression of short-lived TIM-CD4± macrophages in the large intestine

Given our findings in the lung, we next chose to examine the effect of fluoroquinolones on macrophage populations within an additional barrier site. We chose the gut given the recent identification of both resident long-lived and short-lived macrophage populations utilizing CD4 and TIM expression [24]. Gut macrophages negative for both TIM4 and CD4 have a high replenishment rate from blood monocytes, TIM4-CD4+ gut macrophages are turned over more slowly from blood monocytes, while TIM4 + CD4+ macrophages are a locally maintained, gut-resident macrophage population [24].

**Figure 3.**
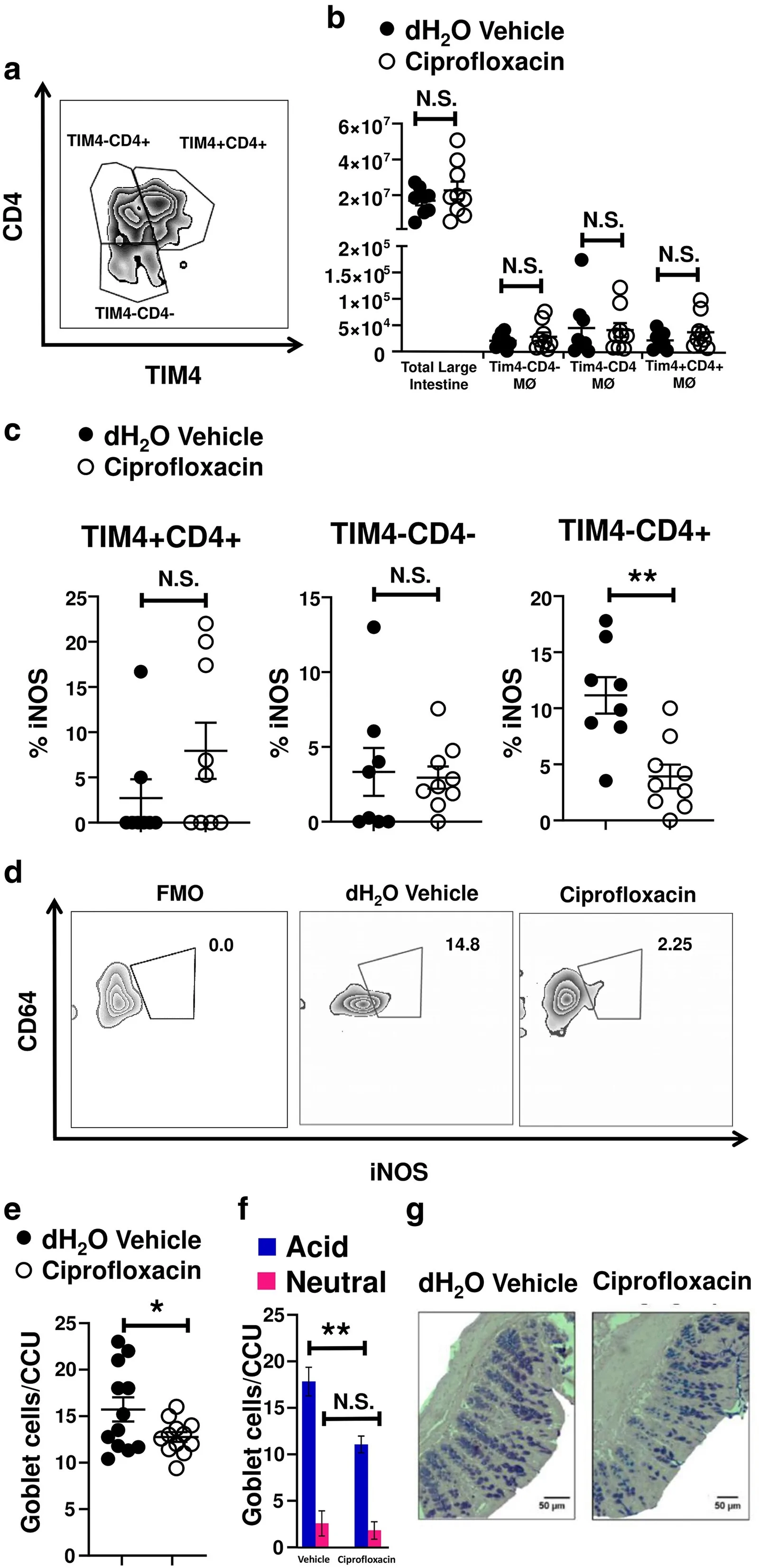
Ciprofloxacin treatment modulates TIM4-CD4+ colonic macrophage iNOS expression and barrier function. C57BL/6 mice were gavaged twice daily with either 100 mg/kg ciprofloxacin or dH_2_O mock dose for 14 days. (a) Identification of TIM4CD4 colonic populations via flow cytometry and total cellularity of colon and macrophage populations via flow cytometry in ciprofloxacin-treated animals (b). Proportion of TIM4CD4 macrophage populations and representative flow cytometry plots of TIM4-CD4+ macrophages in the colon expressing iNOS in ciprofloxacin-treated animals (c and d). Number of colonic goblet cells via PAS/Schiffs staining (e), mucin acidity (f), and representative histology images (g) following harvest at 14 days of treatment. Data (*n* = 8–12 mice per group) are mean ± SEM from three independent experiments performed. *P < 0.05; **P < 0.01; ***P < 0.005; N.S., not significant via Mann–Whitney (b and c), Student’s *t*-test (e) and *ANOVA* followed by Tukey’s multiple comparison test (f) for indicated comparisons between groups.

Ciprofloxacin did not alter the abundance of neutrophil, eosinophil, or DC populations (Supplementary Figure S3a), nor did treatment influence total cellularity or macrophage TIM4CD4 subsets with equivalent numbers in treated animals as compared to vehicle controls (Fig. 3a and b). Examining the expression of iNOS within the tissue-resident TIM4 + CD4+ macrophage population, we saw no alteration in ciprofloxacin-treated groups as compared to dH_2_O-dosed controls (Fig. 3c). The highly replenished TIM4-CD4− population also had comparable iNOS expression between experimental groups; however, the TIM4-CD4+ population had significantly reduced iNOS expression following ciprofloxacin treatment as compared to vehicle controls (Fig. 3c and d). Similar to our lung analysis, ciprofloxacin did not alter colonic immune cell iNOS expression in neutrophil, eosinophil, DC, and B-cell populations as compared to vehicle controls (Supplementary Figure S3b). This antibiotic-induced alteration of iNOS expression exclusively in the TIM4-CD4+ macrophage population was again not observed during treatment with the tetracycline doxycycline but was reproduced following levofloxacin treatment as compared to vehicle controls (Supplementary Figure S3c).

We next examined if this altered pro-inflammatory macrophage phenotype was associated with any intestinal barrier pathology via histological analysis. There was no sign of crypt hyperplasia, an indicator of inflammation, with equivalent crypt depth in both the vehicle- and ciprofloxacin-treated animals (Supplementary Figure S3d). Upon examining goblet cells, it was clear that ciprofloxacin treatment produced a significant reduction in total goblet cell number as compared to vehicle-treated animals (Fig. 3e and g), via a specific reduction in acid mucin containing goblet cells as opposed to those that are neutrally stained (Fig. 3f and g). This intestinal phenotype was mirrored in levofloxacin-treated animals but not doxycycline-treated groups (Supplementary Figure S3e and f).

Collectively, these data indicate fluoroquinolone treatment results in altered gut barrier function and underlying innate macrophage function, similarly to the lung, in a non-resident macrophage population.

### Flouroquinolones directly modulates macrophage phenotype in developing BMDMs

To further explore the observed modulation of macrophage polarization by fluoroquinolones and the altered effects seen in the microbe-rich colon versus lung, we utilized the BMDM system to detect any microbial-independent mechanisms of levofloxacin-induced polarization. This reductionist, *ex vivo* system would allow the observation of any direct actions of levofloxacin on matured macrophages as well as actions on macrophage precursors in the bone marrow.

On examining the purity of the BMDM population in the absence of antibiotics, usually present in most culture systems, we did not see any inhibition in development of macrophages as the high (>96%) purity was seen in all experiments (Supplementary Figure S4a and b). Furthermore, the level of macrophage purity was not altered following overnight incubation with levofloxacin (Supplementary Figure S4a and b). We next examined if levofloxacin was able to drive matured macrophage iNOS expression alone, i.e. in the absence of polarisers such as IFNγ. We found no significant change in iNOS expression in matured macrophages following overnight levofloxacin treatment as compared to the vehicle controls (Supplementary Figure S4c). We next examined the influence of levofloxacin on mature macrophage iNOS expression in the presence of the polarizing cytokine IFNγ. As expected, when BMDMs were stimulated with IFNγ, iNOS expression increased (Supplementary Figure S4c and d). However, addition of levofloxacin with IFNγ did not affect the extent to which BMDMs expressed iNOS as compared to the vehicle controls (Supplementary Figure S4d). To further test this finding, we modulated the level of IFNγ stimulation on our matured BMDMs. Although we saw an increase in iNOS expression in relation to the level of IFNγ stimulation, there was no difference with/without levofloxacin at each IFNγ concentration examined (Supplementary Figure S4e and f). Moreover, modifying the dose of levofloxacin added to the matured macrophages did not influence the expression of iNOS as compared to vehicle-treated cells (Supplementary Figure S4g) Collectively, these data indicate that mature BMDMs purity and potential for iNOS expression is unaltered by the direct action of levofloxacin at the examined doses.

**Figure 4.**
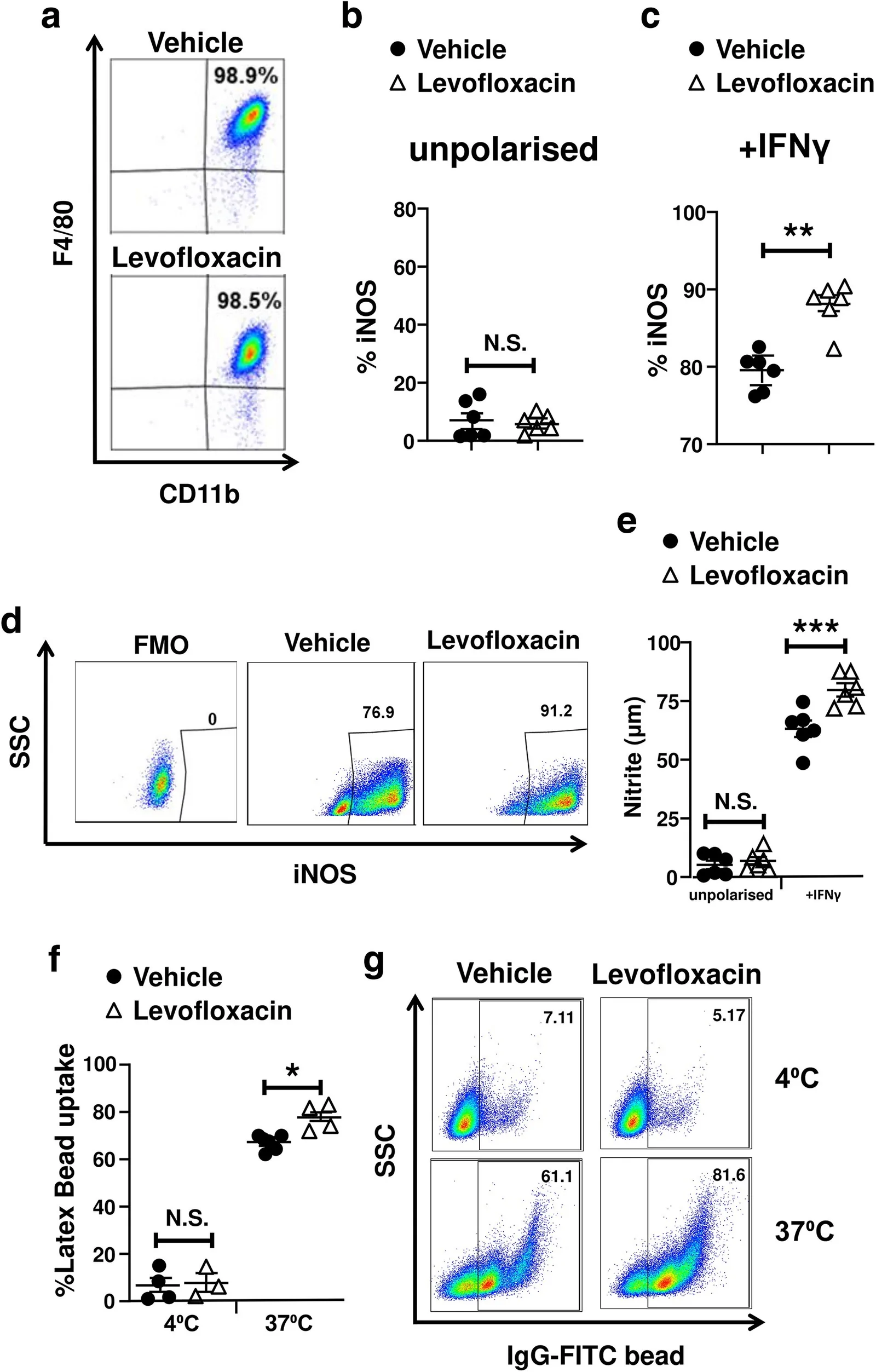
Levofloxacin directly increases iNOS expression and phagocytic ability during BMDM development. Bone marrow cells were isolated from C57BL/6 mice and incubated with the addition of 20 ng/ml M-CSF with/without 30 µM levofloxacin to drive macrophage development and purity was assessed via flow cytometry after 7 days (*a*). iNOS expression was then directly assessed via flow cytometry (b) or following 20 ng/ml IFNγ polarization for 24 hours (c and d). Nitric oxide supernatants levels via Greiss assay (e). Data (*n* = 6) are from three independent experiments performed. After 8 days, with/without 30 µM levofloxacin, mature macrophage cells were incubated with IgG-FITC labelled latex beads for 60 minutes at 37°C, 5% CO_2_ or at 4°C and percentage latex bead uptake by macrophages (f) and representative flow cytometry plots (g) showing uptake. Data (*n* = 4–5) are mean ± SEM from three independent experiments performed. *P < 0.05; **P < 0.01; ***P < 0.005; N.S., not significant via Student’s t-test (b and c) or ANOVA followed by Tukey’s multiple comparison test (e and f) for indicated comparisons between groups.

Our lung and gut *in vivo* macrophage fluoroquinolone data, although contradictory in driving iNOS expression, indicated that shorter-lived monocyte-derived macrophage populations were modulated by the fluoroquinolone in both tissues (Figs 1–3). Given that circulating monocytes are recruited from the bone marrow to specific sites of inflammation and mature to form shorter-lived monocyte-derived macrophages [25], we decided to focus on the effects levofloxacin could have on bone marrow cells if present during their maturation to macrophages. This was achieved by adding levofloxacin to bone marrow cells immediately after their extraction and throughout their 7-day developing incubation. The absence or inclusion of levofloxacin from this early timepoint did not alter the purity of the developing BMDM culture (Fig. 4a) with >97% purity seen in all experiments. Although we saw no significant increase in baseline iNOS expression when levofloxacin was added to maturing BMDMs (Fig. 4b), we saw a significant increase upon the inclusion of polarizing IFNγ, with levofloxacin treatment having significantly more iNOS expression as compared to vehicle controls (Fig. 4c and d). These findings were again recapitulated during ciprofloxacin experiments, with no difference in iNOS baseline expression in unpolarized cells but an increase of iNOS seen in ciprofloxacin-treated versus control BMDMs when added during macrophage development (Supplementary Figure S4h). We confirmed iNOS expression functionality via measuring its stable end product nitrite via the Greiss assay, with levofloxacin again producing a significant increase in nitric oxide only in IFNγ-stimulated cells as compared to vehicle controls (Fig. 4e). Given the increased iNOS expression of macrophages via levofloxacin, we next analysed phagocytosis as a key measure of macrophage function. Reduced phagocytic function has been shown as an indicator of both dysbiosis and metabolite-driven macrophage modulation following antibiotic treatment (Yang et al. 2017 [13], Lankelma et al. 2017 [11]). To determine if levofloxacin treatment of our *ex vivo* BMDM cultures also had this effect, we utilized a phagocytosis assay using latex beads coated with fluorescently labelled IgG. BMDMs were again generated with levofloxacin treatment during maturation and were incubated with beads for 60 minutes at 4°C or 37°C. Although there was minimal bead uptake at 4°C, with comparable phagocytosis between groups, at 37°C levofloxacin caused a significant increase in phagocytosis versus vehicle control (Fig. 4e and f), suggesting a direct modulation of overall macrophage phenotype via levofloxacin.

Collectively, these data suggest fluoroquinolones may have a role in ‘priming’ monocytes influencing developing macrophage function.

### Fluoroquinolones drive macrophage mitochondrial hyperpolarization

**Figure 5.**
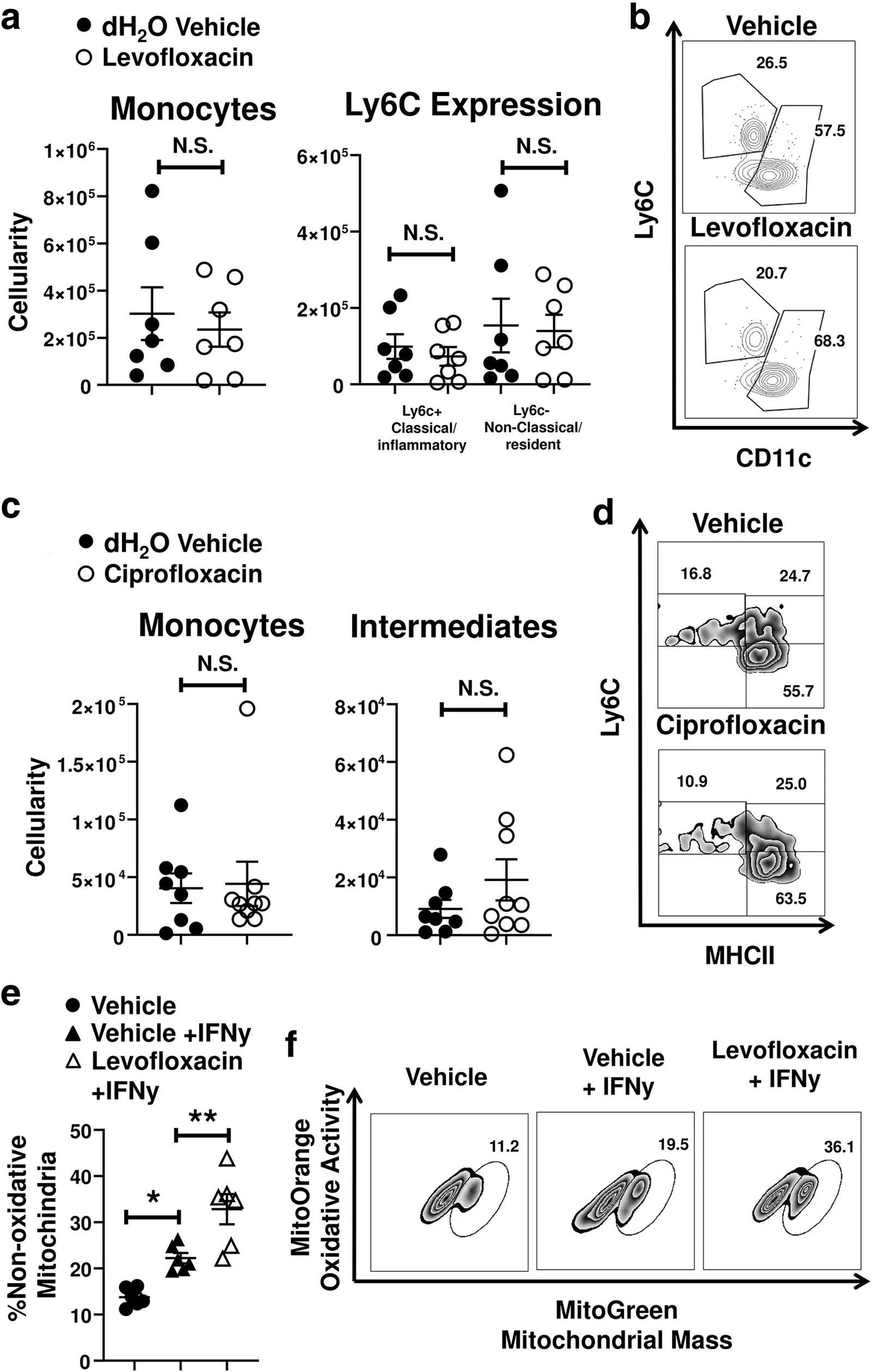
Fluoroquinolones induce mitochondrial hyperpolarization. C57BL/6 mice were gavaged twice daily with either 100 mg/kg levofloxacin or ciprofloxacin or vehicle mock dose for 14 days. Total cellularity of lung monocyte populations (a) and representative flow cytometry plots (b) of Ly6C expressing subsets. Monocyte and intermediate populations in colon (c) and representative flow cytometry plots (d) of MHCII and Ly6C expression of ‘monocyte waterfall’. Data (*n* = 7–9 mice per group) are mean ± SEM from three independent experiments performed. Bone marrow cells were isolated from C57BL/6 mice and matured into macrophages via the addition of 20 ng/ml M-CSF with/without 30 µM levofloxacin during development and IFNy polarization as indicated prior to macrophages being stained with MitoTracker Green FM and MitoTracker Orange CMTMRos with the percentage of non-oxidative mitochondria determined via flow cytometry (e) and representative plots (f). Data (*n* = 6) are mean ± SEM from three independent experiments performed. *P < 0.05; **P < 0.01; ***P < 0.005; N.S., not significant via, not significant via Kruskal–Wallace followed by Dunn’s multiple comparison test (a), Mann–Whitney test (c) or ANOVA followed by Tukey’s multiple comparison test (e) for indicated comparisons between groups.

Given our findings that fluoroquinolones may have a role in ‘priming’ bone marrow cells prior to their development into macrophages, and that in both the lung and gut only shorter-lived monocyte-derived macrophage populations had alterations in iNOS expression, we first assessed if *in vivo* fluoroquinolone treatment had altered monocyte populations in each tissue. Fluoroquinolone treatment did not alter monocyte numbers in the lung of levofloxacin or ciprofloxacin-treated animals as compared to vehicle controls (Fig. 5a and Supplementary Figure S5a respectively). Furthermore, examining monocytes expression of Ly6C to distinguish inflammatory and resident monocytes populations [26] also demonstrated no difference in levofloxacin-treated animals as compared to vehicle controls (Fig. 5a and b). We next examined the monocytes within the colons of ciprofloxacin-treated animals, utilizing Ly6C and MHCII expression to examine the monocyte to macrophage ‘waterfall’ [27]. Both monocyte and intermediate populations were comparable in ciprofloxacin-treated animals as compared to vehicle controls (Fig. 5c and d). Collectively, these data indicate that fluoroquinolone treatment did not alter monocyte or intermediate population within the lung or colon.

We next assessed potential mechanisms of how fluoroquinolones could be directly modulating macrophage development at an intrinsic level. We first sought to rule out the possibility that fluoroquinolones were cytotoxic at the experimental concentrations used *in vitro*. To investigate this a lactate dehydrogenase (LDH) assay was performed to measure enzyme release by damage to the plasma membrane, thus indicating cytotoxicity. Neither ciprofloxacin or levofloxacin produced any cytotoxicity at any of the experimental concentrations used, while forcibly lysed cells produced the expected increases in LDH acting as a positive control (Supplementary Figure S5b). Certain antibiotics are known to be particularly acidic with *in vivo* concentrations and given macrophage polarization can be influenced by pH [28], we wanted to determine if this was responsible in our *ex vivo* BMDM cultures. The pH of BMDM culture media was measured with and without fluoroquinolones, with no significant difference between pH observed in any of the conditions, and delivery times, as compared to non-antibiotic controls (Supplementary Figure S5c). Collectively, these data show that fluoroquinolone effects on macrophage phenotype *in vitro* are independent of cytotoxicity and pH.

Interestingly, macrophage inflammatory polarization, including iNOS expression, is well established to involve mitochondrial hyperpolarization known to drive mitochondrial repurposing from ATP synthesis to ROS production (Mills et al. 2016 [15]). We therefore hypothesized if fluoroquinolone-induced mitochondrial membrane potential alterations could be linked to our observations of increased inflammatory polarization and phagocytosis.

To detect direct fluroquinolone alterations on developing macrophages, we returned to the BMDM system and examined mitochondrial function utilizing dual MitoTracker staining to quantify overall mitochondrial mass and oxidative activity via flow cytometry, as previously described [29]. As expected, cells treated with IFNγ had a significant increase in non-oxidative macrophages as compared to vehicle controls (Fig. 5e and f). Interestingly, the addition of either levofloxacin (Fig. 5e and f) or ciprofloxacin (Supplementary Figure S5d), but not doxycycline (Supplementary Figure S5d), significantly increased the percentage of non-oxidative mitochondria following IFNγ stimulation as compared to vehicle polarized cells alone. Importantly, this hyperpolarization was also uncoupled from iNOS expression, with hyperpolarization also occurring in levofloxacin-treated cells prior to IFNγ treatment and iNOS expression (Supplementary Figure S5e).

Collectively, these findings indicate fluoroquinolones directly cause mitochondrial hyperpolarization in *ex vivo* developed macrophages suggesting a potential mechanism for priming macrophage inflammatory polarization.

## Discussion

In this study, naïve C57BL/6 mice were treated with a regime of fluoroquinolones over a 2-week period, and innate immune populations were examined at likely barrier sites of potential bacterial infection. We first focussed on the lung, observing mild pathology at the macroscopic level in terms of increased lung alveolar space in levofloxacin-treated animals. Recently, fluoroquinolones have been demonstrated to cause serious adverse effects through potential collagen toxicity [30], particularly collagen types I and III, by upregulating the expression of matrix metalloproteases [31–33]. Indeed, the most widely reported adverse effects of fluoroquinolones are tendon-related, such as tendonitis or even tendon rupture, and other severe collagen-associated adverse effects such as retinal detachment and aortic aneurysms [34, 35]. However, a recent study ruled out any association of spontaneous pneumothorax with fluoroquinolones, with data strongly suggesting the role of the underlying infection rather than antibiotic choice was responsible [36]. Indeed, we did not see any pathology in the lung of ciprofloxacin-treated animals, but this may also be due to the reduced penetration of ciprofloxacin as compared to levofloxacin in lung [37].

We next examined innate immune populations and observed no alteration in neutrophils, eosinophils or DC numbers in lung or colon barrier tissues, or iNOS expression in these immune lung populations. Neutrophils have been shown to actively internalize fluoroquinolones [38] enhancing bactericidal mechanism, but only rare individual reports of neutropenia during moxifloxacin treatment [39] and fluoroquinolone-induced eosinophilia exist [40]. We could find no reports of fluoroquinolone mediated immunomodulatory of dendritic cell, although ciprofloxacin-resistant bacteria have been shown to reside in caecal draining dendritic cells thought to allow recalcitrance to antibiotics treatment [41]. Our macroscopic examination of colonic tissues demonstrated fluoroquinolone specific depletion of neutral mucin expressing goblet cells, although we observed no alteration in lung tissues. Prophylactic ciprofloxacin use in the fish industry, has been observed to also decrease goblet cells in the intestine of ayu, with associated microbial dysbiosis and increased anti-inflammatory cytokine release seen [42]. Indeed, a ‘microbiome-goblet cell protection’ model in which antibiotic-induced dysbiosis rather than the direct effect of antibiotics on goblet cells is suggested [43]. We saw no changes in other immune populations in terms of iNOS expression, however although we did not assess intestinal epithelial iNOS levels, it is of note that reduced Paneth cell iNOS expression, as seen in our fluoroquinolone-treated intestinal macrophage population, results in microbiota-independent goblet cell hypoplasia [44]. Further organoid studies would allow the assessment of direct fluoroquinolone effects on epithelial goblet cell differentiation.

As highlighted, our main findings on fluoroquinolone immunomodulatory effects were on cellular subsets of macrophage populations within both the lung and colon. This was consistent with ciprofloxacin and levofloxacin but not observed during doxycycline treatment, the unique structural features of fluoroquinolones have been suggested as responsible for their immunomodulatory properties [45]. Moreover, doxycycline has previously been shown to have numerous direct anti-inflammatory properties [46], including reducing iNOS mRNA levels in a lung epithelial cell line [47]. Doxycycline treatment has been shown to both decrease [48] and increase [49] macrophage iNOS expression; however, we saw no changes in iNOS expression in any lung or gut macrophage population examined potentially due to our prophylactic treatment regime and extensive subsetting. Murine macrophages are dispersed throughout the body, composing phenotypically distinct subtypes, not only highly specialized for, but also influenced by the environment in which they are located [50–52]. Alveolar macrophages are located within the airway and appear to be largely homogenous [53], while interstitial tissue (also known as perivascular) macrophages are a much more heterogenic population. These populations can be identified by expression of CD64 at the macrophage level and subdivided by CD11c, CD11b expression [54–56], while interstitial populations can be subdivided further via the expression of the mannose receptor CD206 [23, 26, 54]. Utilizing these cellular markers, we demonstrated although fluoroquinolones did not alter any population cellularity, the CD206− interstitial population uniquely had increased iNOS expression, potentially suggesting increased pathogen clearance potential. The reason for this distinct macrophage modulation may be due to their location, as CD206 expression is thought to distinguish the tissue niche, with CD206− found within the tissue adjacent to the alveolar associated with nerves, while the CD206 population occupies the perivascular niche [23, 26]. It is important to point out that our tissue digestion and flow cytometry analysis brings the caveat of potentially missing rare cell populations and cellular positioning. Future studies utilizing immunofluorescence, 3D imaging and ultimately spatial transcriptomics would further delineate the importance of the tissue niche in our observed distinct macrophage subset modulation.

Another potential reasoning for our findings is ontogeny, with alveolar macrophages in adult mice mainly deriving from foetal liver progenitors, with the ability to maintain themselves via self-renewal [57–60], although alveolar macrophages may require replenishment from bone-marrow-derived monocytes over the entire life course [61]. Interstitial lung macrophages have an initial yolk sac progenitor [62, 63], but these are subsequently replaced first by foetal liver derived macrophages and then continually by bone-marrow-derived monocytes [23, 62]. Although we saw no difference in cellularity in our macrophage populations that can be associated with inflammation, the modest inflammation observed in our lung histology, would potentially cause macrophage replacement by the known mechanisms of alveolar macrophages local proliferation [61], while interstitial populations are replaced by circulating monocytes [64]. It must be pointed out that CD206+ interstitial monocyte replenishment occurs 2 days prior to the CD206− population in diptheria depletion experiments [65 ], so other factors other than monocyte ontogeny must be at play in our subset iNOS fluoroquinolone effects. The subsets also have unique transcriptional profiles [65] and as previously mentioned tissue niches [23, 26], so further elucidation of their roles may uncover their differing responses to fluoroquinolones.

Our findings in colonic macrophages also indicated alterations in iNOS expression in specific subsets following fluoroquinolone treatment, with iNOS expression reduced in the recently identified TIM4-CD4+ population. In parallel to the lung, TIM4 + CD4+ gut macrophages have been found to be locally maintained, while TIM4-CD4+ macrophages have a slow turnover from blood monocytes, with TIM4-CD4− macrophages possessing a high monocyte-replenishment rate currently attributed to gut macrophages [24]. Although we did not see any alteration in colonic macrophage populations in our individual antibiotic-treated mice, TIM4-CD4+ macrophages were reduced in germ-free studies, while TIM4-CD4− numbers were unaltered, indicating different responses to the microbiota, with transcriptional analysis highlighting heterogeneity as well as tissue niches [24, 66], again pointing to multiple factors which could be influencing responses to fluoroquinolones.

Given our differing findings of iNOS expression in the lung and colon macrophage susbsets, we utilized the BMDM system to assess direct immunomodulatory effects independent of the microbial influence. Interestingly, we saw increased iNOS expression, but only in developing macrophages and not established ones at multiple cytokine and antibiotic concentrations, potentially mirroring our observations of influence on the monocyte-derived *in vivo* macrophage subsets in the lung and colon. Previous studies have demonstrated short incubation times as altering macrophage polarization with stimulation of RAW 264.7 murine macrophages with oxacillin leading to significantly higher iNOS. However, this was not observed on RAW cells alone and pneumococci dependent [67]. Conversely, studies utilizing short timed doses of ofloxacin on murine peritoneal macrophages *ex vivo* increased reactive oxygen species while decreasing iNOS [68]. The bactericidal concentration of quinolones can lead to DNA fragmentation which results in production of superoxide from macrophages [69] again indicating bacteria dependence in iNOS modulation, which was not present in our studies. Moreover, multiple quinolones have been shown to increase super oxide expression dose dependently in rat peritoneal macrophages, but in parallel to our findings, not in neutrophils [70]. Utilizing the standard combination antibiotic treatment for *Mycobacterium tuberculosis* drives potential anti-inflammatory immunomodulatory actions on macrophages *in vitro* [71], demonstrating antibiotic macrophage polarization abilities in the absence of bacteria. Interestingly, in this study, it was suggested that the intermediate status population during monocytes to macrophage differentiation was targeted [71]. This, in combination with our studies, demonstrating levofloxacin-induced effects only on developing and not mature macrophages, again supports the possibility that antibiotics may be more influential on monocytes/intermediate monocytes than macrophages themselves. Phagocytosis assays are commonly used to quantify impact on macrophage immune function, often with a reduction of phagocytosis as a marker of a treatment negatively affecting macrophage function [11]. Yang and Bhargava [13] showed that ciprofloxacin treatment leads to reduced phagocytic engulfing and pathogen killing in macrophages, while multiple quinolones have been shown to reduce the phagocytosis of rat peritoneal macrophages of fluorescein-conjugated *E*. *coli* [70]. This may be explained by our differing methodology, where these studies measured phagocytosis via use of *E. coli* in macrophage cell lines and peritoneal macrophages rather than IgG-coated beads and BMDMs as used here limiting comparability.

We next approached potential mechanisms of how fluoroquinolones could directly alter macrophage function. Fluoroquinolones and doxycycline have been shown to modulate TLR2 and 4 expression, reducing potential inflammatory cytokine production in monocytes and macrophages [72]. The fluoroquinolone antibiotic moxifloxacin has been shown to have immunomodulatory activity through its capacity to increase the secretion of IL-1α and TNF-α by human monocytes in the presence of LPS [73]. In contrast, another fluoroquinolone, trovafloxacin, significantly inhibited secretion of cytokines by monocytes stimulated with LPS [74]. Both studies, however, do indicate that fluoroquinolones do have the potential to influence monocytes *in vitro* in a similar timescale to our own experiments. Recently, experiments on the RAW264.7 macrophage cell line have demonstrated that environmental levels of quinolones, including ciprofloxacin, enhanced phagocytosis, and increased PI3K/Akt, Notch1, JNK, and JAK2/STAT3 signalling pathways to cause the secretion of pro-inflammatory cytokines and induce inflammation [75].

In aiming to examine the potential initiating molecular mechanisms, we turned to several studies that have linked many tendinopathy side effects of fluoroquinolone symptoms in patients to mitochondrial damage, likely due to mitochondria’s evolutionary semblance to prokaryotic cells [76–79]. Moreover, historically trovafloxacin was withdrawn from the global market relatively soon after approval because of resulting serious liver injury, with recent 3D liver toxicity models highlighting mitochondrial ROS formation as an intrinsic toxicity mechanisms, although this was absent in levofloxacin treatment [80]. Kalghatgi and Spina [76] showed how several classes of antibiotic, including fluoroquinolones cause oxidative damage and the overproduction of reactive oxygen species and mitochondrial dysfunction *in vitro* and *in vivo.* Mitochondria are intrinsically linked with macrophages and their function [81]. Pro-inflammatory macrophages, specialized for pathogen killing, alter their metabolism to limit oxidative phosphorylation (OXPHOS) and fatty acid oxidation, both mitochondrial processes. They in turn switch towards a glycolysis-heavy metabolism, in order to rapidly produce ATP to fuel intensive pathogen killing [82]. It therefore stands to reason that any effects of fluoroquinolones on macrophage polarization may well be caused by altered mitochondrial function. Bedaquiline induces a significant metabolic reprogramming of human monocyte-derived resting macrophages, without altering cell viability and increases genes including those associated with lysosome, phagocytic vesicle membrane as well as lipid homeostasis [14]. Therefore, it remains possible that antibiotics may alter macrophage function via subtle effects on mitochondrial function.

We therefore utilized MitoTrackers to quantify both mitochondrial mass and oxidative activity to assess function [29]. As expected, we observed an increase in mitochondrial non-oxidative function following the addition of IFNy to our BMDMs, to similar levels seen by others utilizing LPS stimulation [29]. However, the addition of fluoroquinolones again reduced oxidative mitochondrial function, suggesting an additional shift from OXPHOS to glycolysis, leading to further hyperpolarization of the inner mitochondrial membrane. Mitochondrial hyperpolarization is required for the pro-inflammatory effects of LPS on BMDMs [15]. Indeed, during inflammatory activation ‘glycolytically competent cells’ such as macrophages use significant amounts of the glycolytically generated ATP to drive mitochondrial hyperpolarization and thereby prevent apoptosis [83]. Our findings are supported by the direct dose-dependent inhibition of ciprofloxacin on the measured oxygen consumption rate in the mouse macrophage cell line SV129 [13]. Moreover, autophagy-deficient macrophages demonstrated reduced oxidative mitochondrial function, which drove ROS-dependent pro-inflammatory function [84]. This mechanism may also explain why we observed specific macrophage populations being affected by fluoroquinolone treatment *in vivo*, as it has recently been identified that tissue macrophages differentially use and need OXPHOS in the steady state [85]. Interestingly, within the intestine of pan-antibiotic-treated animals macrophages increased oxygen consumption, while mitochondrial function was improved specifically within the TIM4-CD4+ population [86]. This additional heavy microbial load of the gut versus lung correlates with our reduced iNOS expression within this sole colon macrophage population in fluoroquinolone-treated animals and potentially explains our contrasting gut phenotype from our lung and BMDM experiments.

There is some controversy in terms of the importance of mitochondrial repurposing in driving or being a simple effect of expansive NO production [87]. It should be noted that NO in itself can cause mitochondrial rewiring and is currently being reframed as a cause rather than the effect of macrophage metabolic changes [88]. Other mechanisms could also be responsible; a recent study on trovafloxacin suggests that reactive metabolites of this fluoroquinolone cause the release of damage-associated molecular patterns from hepatocytes that can activate the inflammasome, which would also initiate inflammation [89]; conversely, the inflammasome could also be triggered via antibiotic-induced mitochondrial damage. We should note that we did observe decreased oxidative mitochondrial function in the absence of IFNγ stimulation and, importantly, resulting iNOS expression, indicating a priming that may require the secondary inflammatory licensing of IFNy. Moreover, simply targeting mitochondrial proteins results in mitochondrial hyperpolarization followed by ROS expression as well as increased phagocytosis in monocyte and macrophage human cell lines [90], demonstrating that fluoroquinolone-driven mitochondrial hyperpolarization resulting in a pro-inflammatory macrophage state alone could occur in certain contexts.

In conclusion, we have demonstrated that fluoroquinolones target macrophage iNOS expression in the barrier sites of both the lung and colon. This is macrophage population specific, being observed in shorter-lived monocyte-derived CD206− interstitial lung and TIM-CD4+ colon populations. One potential molecular mechanism by which fluoroquinolones may influence macrophage phenotype is a direct intrinsic effect on developing macrophage mitochondrial hyperpolarization, as this was associated with increased iNOS expression and phagocytosis in *ex vivo* BMDMs. This study emphasises the direct immune-modulating effects of specific antibiotic treatments on macrophage populations that are often essential guards against bacterial infections that antibiotic usage targets. These data therefore highlight the direct immunomodulatory effects of fluoroquinolones, while also highlighting that the *in vivo* setting may provide additional context-specific signals that may prevail these direct effects, which could influence antibiotic selection during therapy.

## Supplementary Material

kyaf018_Supplementary_Data

## Data Availability

The data underlying this article are available in the article and in its online Supplementary material.
