# Supplementary material for: Fluoroquinolones directly drive mitochondrial hyperpolarization and modulate iNOS expression in monocyte-derived macrophage populations
Source: Discov Immunol. 2025 Nov 12;4(1):kyaf018. doi: 10.1093/discim/kyaf018 (PMC12770987; doi:10.1093/discim/kyaf018)
Supplement: kyaf018_Supplementary_Data [file kyaf018_supplementary_data.zip › Supplementary_figure_legends.pdf]

Supplementary Figure 1. Levofloxacin treatment does not alter lung neutrophil, eosinophil and DC numbers or alveolar and interstitial CD206+ macrophage iNOS expression respectively. C57BL/6 mice were gavaged twice daily with either 100mg/kg levofloxacin or dH_2O mock dose for 14 days. Goblet cell numbers/High power field (HPF) of lungs (*A*) via PAS/Schiffs histology following harvest at 14 days post treatment. Total cellularity of lung neutrophil, eosinophil and DCs (*B*) via flow cytometry. Proportion of alveolar *(C)* and interstitial CD206+ *(D)* macrophages in the lung expressing iNOS. Data (n=7 mice per group) are mean ± S.E.M from three independent experiments performed. *, P<0.05; **, P<0.01; ***, P<0.005,; N.S. , not significant via *ANOVA* followed by Dunnetts's multiple comparison test *(A, B and C)* or Kruskal-Wallace followed by Dunn's multiple comparison test *(D)* for indicated comparisons between groups.

Supplementary Figure 2. Ciprofloxacin and doxycycline treatment do not influence lung macrophage numbers or alveolar and interstitial CD206+ macrophage iNOS expression respectively. C57BL/6 mice were gavaged twice daily with either 100mg/kg levofloxacin or doxycycline or dH_2O/PBS mock dose for 14 days. Total cellularity of lung and macrophage populations via flow cytometry in ciprofloxacin and doxycycline treated animals (*A and E*). Proportion of alveolar and interstitial CD206+ macrophages in the lung expressing iNOS in ciprofloxacin and doxycycline treated animals (*B,C and F,G*). Proportion of lung neutrophils, eosinophils, DCs and B-cells expressing iNOS in ciprofloxacin treated animals *(D)*. Data (n=7 mice per group) are mean ± S.E.M from three independent experiments performed. *, P<0.05; **, P<0.01; ***, P<0.005,; N.S. , not significant via Kruskal-Wallace followed by Dunn's multiple comparison test *(A, C)*, *ANOVA* followed by Dunnetts's multiple comparison test *(B)*, Mann-Whitney *(D, E)*, or Student's t-test *(F and G)* for indicated comparisons between groups.

Supp. Figure 3. Levofloxacin, but not doxycycline, modulates TIM4-CD4+ colonic macrophage iNOS expression and barrier function. C57BL/6 mice were gavaged twice daily with either 100mg/kg levofloxacin/ciprofloxacin or doxycycline or dH_2O/PBS mock dose

for 14 days. Total cellularity of colonic neutrophil, eosinophil and DCs in ciprofloxacin treated and vehicle control mice (*A*) via flow cytometry. Proportion of colonic neutrophils, eosinophils, DCs and B-cells expressing iNOS in ciprofloxacin treated animals *(B)*. Representative flow plots of TIM4-CD4+ macrophage populations in the colon expressing iNOS in indicated animals (*C*). Crypt depth in ciprofloxacin and vehicle treated animals *(D)*. Number of colonic goblet cells via PAS/Schiffs staining *(E)* and mucin acidity and representative histology images (*F*) following harvest at 14 days of treatment. Data (n=5-12 mice per group) are mean ± S.E.M from three independent experiments performed. *, P<0.05; **, P<0.01; ***, P<0.005,; N.S. , not significant via Mann-Whitney *(A, B)*, Student's t-test *(D and E)* and *ANOVA* followed by Tukey's multiple comparison test *(F)* for indicated comparisons between groups.

Supplementary Figure 4. Levofloxacin does not affect iNOS expression in matured BMDMs. Bone marrow cells were isolated from C57BL/6 mice and matured into macrophages via the addition of 20ng/ml M-CSF. After 7 days BMDMs were treated with 30µM levofloxacin and purity was assessed via flow cytometry (*A and B*). iNOS expression was then assessed via flow cytometry without (*C*) and with (*D*) 20ng/ml IFNγ polarisation for 24hrs. Data (n=5-6) are mean ± S.E.M from three independent experiments performed. iNOS expression via flow cytometry in BMDMs assessed in 5 and 40ng/ml of IFNγ *(E and F)* and 10 and 40µM levofloxacin. Data (n=3-4) are mean ± S.E.M from three independent experiments performed. Bone marrow cells were isolated from C57BL/6 mice and incubated with the addition of 20ng/ml M-CSF with/without 30µM ciprofloxacin to drive macrophage iNOS expression was then directly assessed via flow cytometry or following 20ng/ml IFNγ polarisation for 24hrs, representative flow cytometry plots *(H)*. Data (n=5-6) are mean ± S.E.M from three independent experiments performed. *, P<0.05; **, P<0.01; ***, P<0.005,; N.S. , , not significant via Student's t-test *(A, B, C)* or *ANOVA* followed by Tukey's *(E)* or Dunnetts's *(G)* multiple comparison test for indicated comparisons between groups.

Supplementary Figure 5. Fluoroquinolones do not induce cytotoxicity or alter pH to BMDMs, but do induce mitochondrial hyperpolarisation prior to IFNy induced iNOS expression. C57BL/6 mice were gavaged twice daily with 100mg/kg ciprofloxacin or mock dose for 14 days. Total monocyte population of the lung via flow cytometry (*A*). Data (n=7 mice per group) are mean ± S.E.M from three independent experiments performed. Bone marrow cells were isolated from C57BL/6 mice and matured into macrophages via the addition of 20ng/ml M-CSF for 7 days. Macrophages were treated with a range of ciprofloxacin and levofloxacin concentrations from 0.1-100µg/ml, including positive lysis and spontaneous lysis controls, and a lactate dehydrogenase assay performed *(B)*. Data (n=3) are mean ± S.E.M from three independent experiments performed. Bone marrow cells were isolated from C57BL/6 mice and matured into macrophages via the addition of 20ng/ml M-CSF for 7 days with /without 30µM ciprofloxacin or levofloxacin during development or on maturation as indicated. Media was assessed for pH *(C)* using a benchtop pH meter. Data (n=2) are mean ± S.E.M from two independent experiments performed. Bone marrow cells were isolated from C57BL/6 mice and matured into macrophages via the addition of 20ng/ml M-CSF for 7 days with /without 30µM ciprofloxacin, levofloxacin or doxycycline during development and macrophages were stained with MitoTracker Green FM and MitoTracker Orange CMTMRos with the percentage of non-oxidative mitochondria determined via flow cytometry in IFNy polarised *(D)* or non-polarised conditions *(E)* as indicated. Data (n=5-6) are mean ± S.E.M from three independent experiments performed. *, P<0.05; **, P<0.01; ***, P<0.005,; N.S. , not significant via Kruskal-Wallace followed by Dunn's multiple comparison test *(A)*, *ANOVA* followed by Tukey's multiple comparison test *(B, C, D)* or via Student's t-test *(E)* for indicated comparisons between groups.
