# Supplementary figures and images for: Fluoroquinolones directly drive mitochondrial hyperpolarization and modulate iNOS expression in monocyte-derived macrophage populations
Source: Discov Immunol. 2025 Nov 12;4(1):kyaf018. doi: 10.1093/discim/kyaf018 (PMC12770987; doi:10.1093/discim/kyaf018)

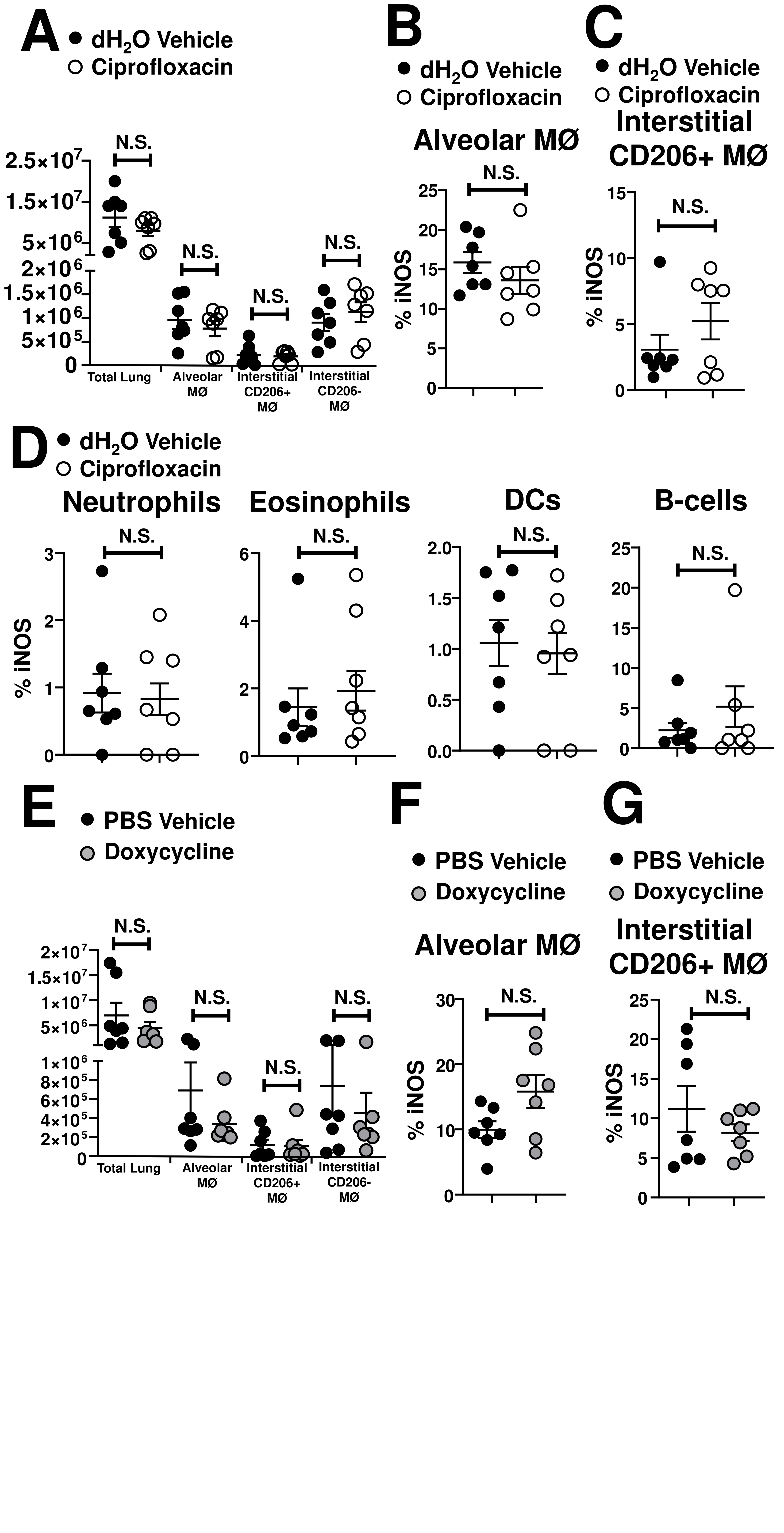

Supplement: kyaf018_Supplementary_Data [file kyaf018_supplementary_data.zip › Supp_Fig_2[1].tif]

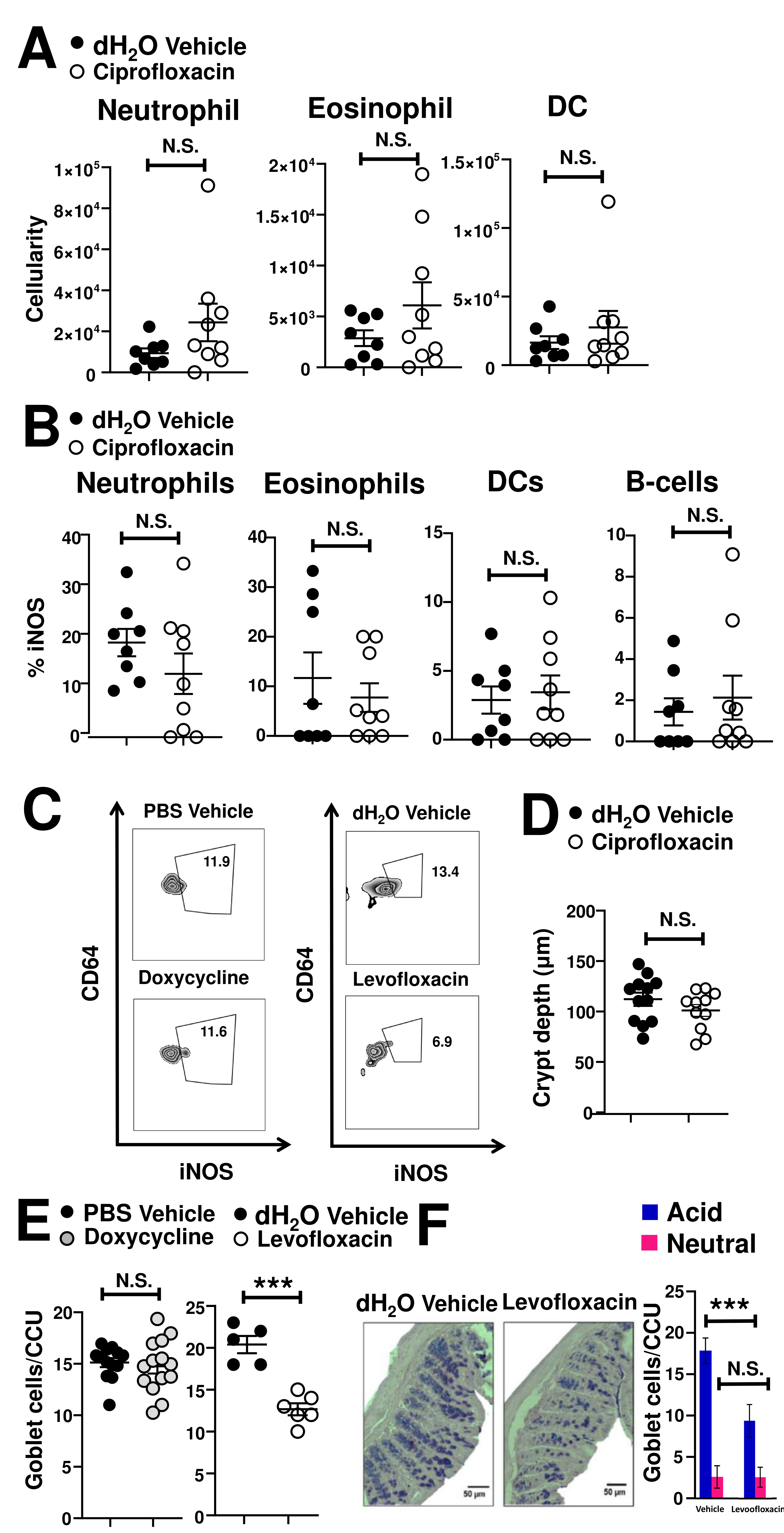

Supplement: kyaf018_Supplementary_Data [file kyaf018_supplementary_data.zip › Supp_Fig_3[1].tif]

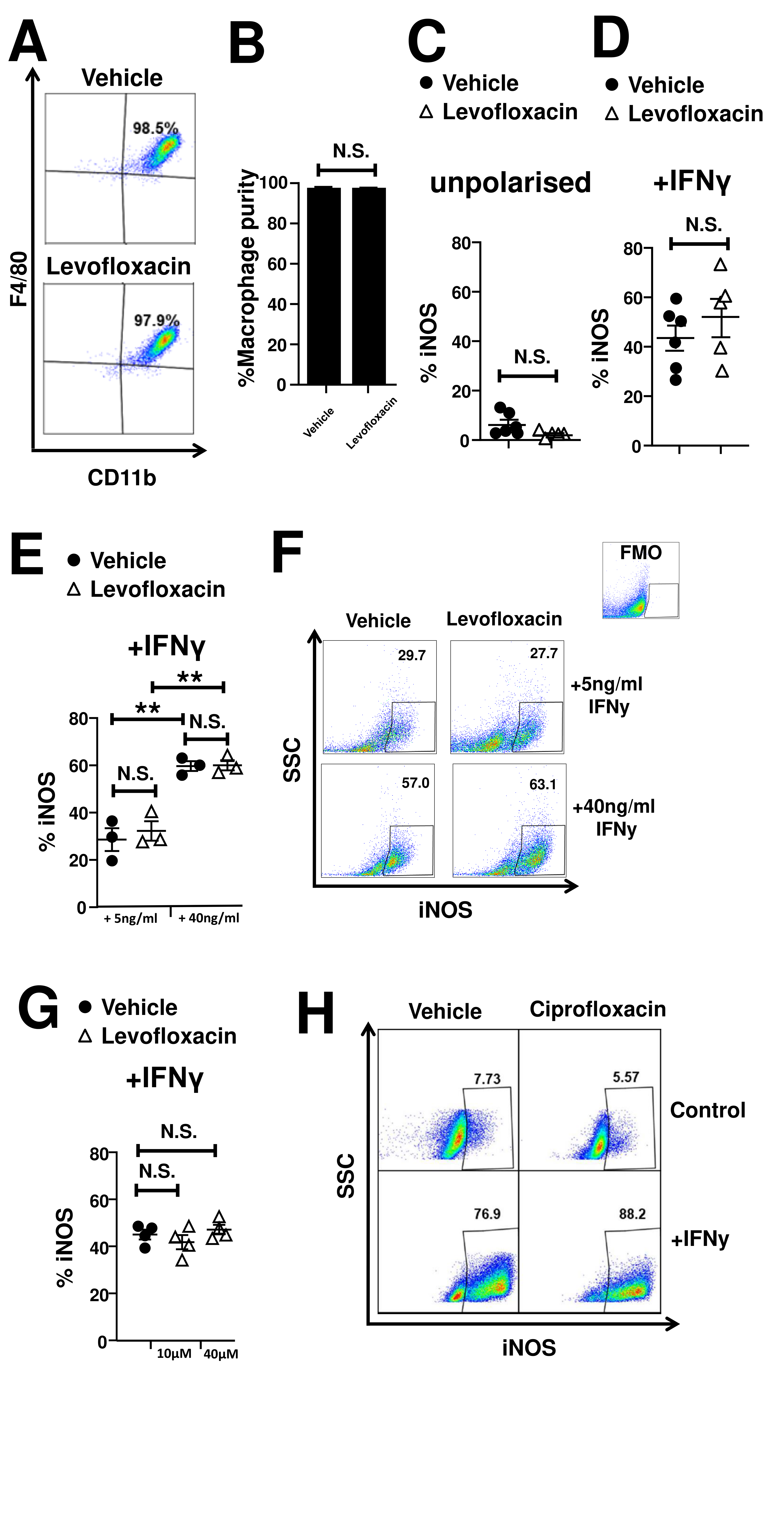

Supplement: kyaf018_Supplementary_Data [file kyaf018_supplementary_data.zip › Supp_Fig_4[1].tif]

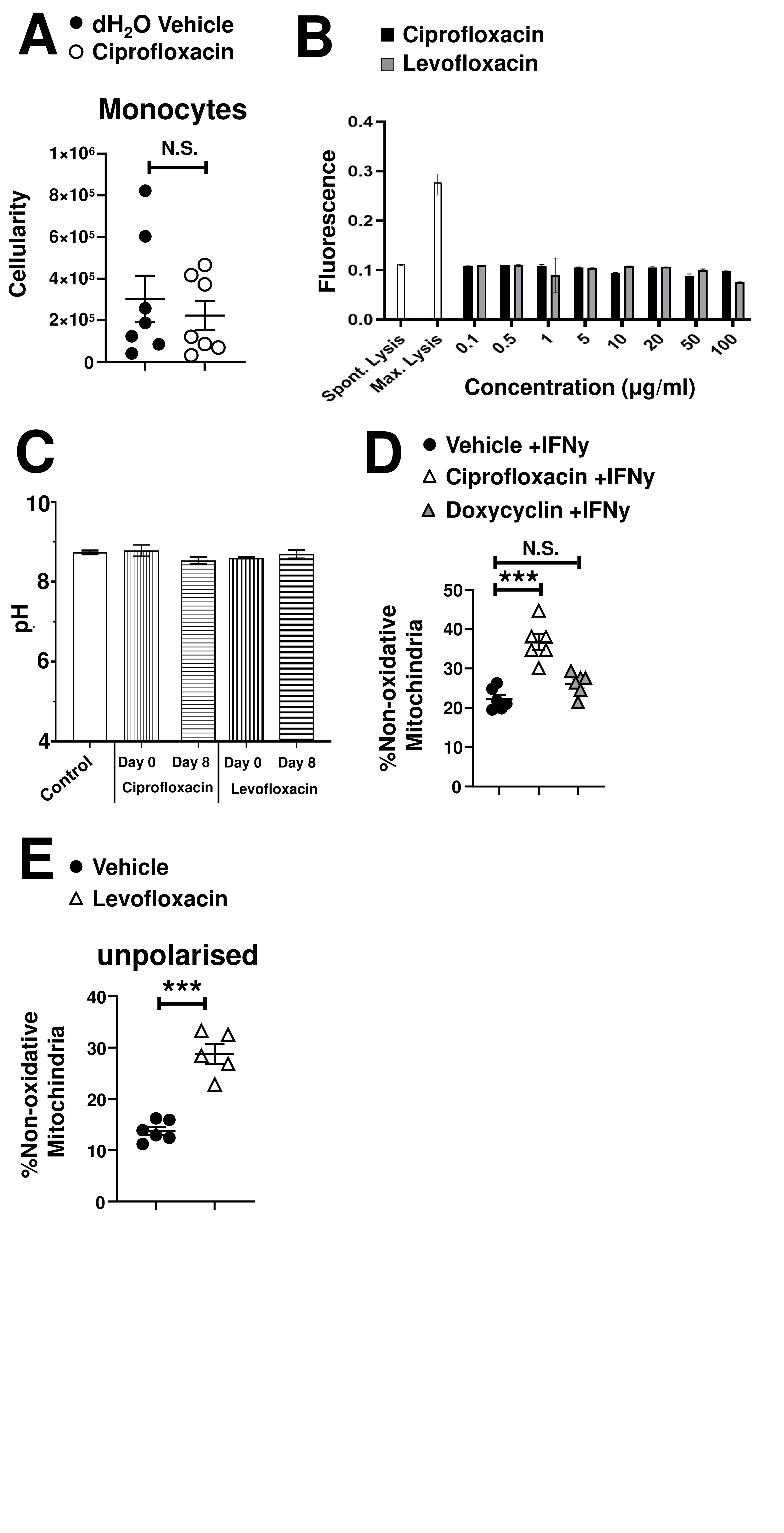

Supplement: kyaf018_Supplementary_Data [file kyaf018_supplementary_data.zip › Supp_Fig_5[1].tif]
